# Molecular Characterization, Structural Modeling, and Evaluation of Antimicrobial Activity of Basrai Thaumatin-Like Protein against Fungal Infection

**DOI:** 10.1155/2017/5046451

**Published:** 2017-08-10

**Authors:** Nusrat Yasmin, Mahjabeen Saleem, Mamoona Naz, Roquyya Gul, Hafiz Muzzammel Rehman

**Affiliations:** ^1^Institute of Biochemistry and Biotechnology, University of the Punjab, Lahore 54590, Pakistan; ^2^The University of Lahore, Lahore 54590, Pakistan

## Abstract

A thaumatin-like protein gene from Basrai banana was cloned and expressed in* Escherichia coli*. Amplified gene product was cloned into pTZ57R/T vector and subcloned into expression vector pET22b(+) and resulting pET22b-basrai TLP construct was introduced into* E. coli* BL21. Maximum protein expression was obtained at 0.7 mM IPTG concentration after 6 hours at 37°C. Western blot analysis showed the presence of approximately 20 kDa protein in induced cells. Basrai antifungal TLP was tried as pharmacological agent against fungal disease. Independently Basrai antifungal protein and amphotericin B exhibited their antifungal activity against* A. fumigatus*; however combined effect of both agents maximized activity against the pathogen. Docking studies were performed to evaluate the antimicrobial potential of TLP against* A. fumigatus* by probing binding pattern of antifungal protein with plasma membrane ergosterol of targeted fungal strain. Ice crystallization primarily damages frozen food items; however addition of antifreeze proteins limits the growth of ice crystal in frozen foods. The potential of Basrai TLP protein, as an antifreezing agent, in controlling the ice crystal formation in frozen yogurt was also studied. The scope of this study ranges from cost effective production of pharmaceutics to antifreezing and food preserving agent as well as other real life applications.

## 1. Introduction

According to Selitrennikoff [1], in every ecological system about 250,000 fungi are generally distributed and certain microorganisms are able to produce severe damaging effect on quality as well as production of important crop plants. During evolutionary process, plants adapted to gradual climatic changes and they acquired potential defense mechanisms, including low molecular weight compounds, proteins, and peptides exhibiting antimicrobial activities. The pathogenesis-related (PR) proteins were first described by Van Loon and Van Kammen [2] after observing the accumulation of numerous proteins in tobacco plants when infected with microbial agents like tobacco mosaic virus (TMV).

From various dicotyledon and monocotyledon plant species, PR5 proteins have been isolated and characterized [3, 4]. Protein belonging to this group exhibited antifungal activity against a large number of various fungal pathogens; however their biological functions are not still recognized [3–5]. Proteins of PR5 group have been characterized from different plant sources such as corn, soybeans, rice, and wheat [6, 7]. PR5 proteins share their sequence and structural similarity with the sweet tasting protein from* Thaumatococcus danielli* (thaumatin) but do not exhibit any sweet property and hence are recognized as thaumatin-like (TL) proteins [8, 9].

On the basis of their molecular mass, TLPs are categorized in two groups: one group of proteins with molecular weight ranging from 22 to 26 kDa whereas the other group includes proteins of 18 kDa or less. First group proteins usually accumulate in cell vacuoles while proteins from the second group are mostly found extracellularly [10]. TLPs of the larger group comprise 16 cysteine amino acid residues resulting in the formation of 8 disulfide bridges, whereas 10 cysteine residues found among the proteins of smaller group form 5 disulfide bonds. The disulfide bridges are responsible for their resistance against protease enzymes and pH or heat induced denaturation. TLPs have also been discovered in animals, more specifically in nematodes and insects [11], and in fungi [12]. TLPs might play a defense role against pathogens in these organisms just alike in plants.

Thaumatin-like proteins are not restricted to vegetative tissues but have also been identified in fruits of different dicots. The literature revealed that cherries, tomatoes, and grapes accumulate large quantities of PR-5 proteins during ripening [13].

The current study describes the cloning and expression of pathogenesis-related thaumatin-like antifungal protein (Basrai TLP) from Basrai banana and its role as antifungal, therapeutic, and antifreezing agent.

## 2. Materials and Methods

### 2.1. Isolation of Genomic DNA Banana Pulp

Genomic DNA was extracted by the method of Sangeetha et al. [14]. One gram of banana pulp was pulverized to a fine powder with liquid nitrogen in a sterilized mortar and pestle and then 10 ml hot CTAB buffer containing 100 mM Tris-Cl (pH 8.0), 20 mM EDTA (pH 8.0), 1.4 M NaCl, 2.0% CTAB, and 1% polyvinylpyrrolidone (PVP) was added. The sample was incubated at 65°C in a shaking water bath for 30 minutes. After cooling, equal volume of freshly prepared chloroform : isoamyl alcohol in the ratio of 24 : 1 was added and centrifuged 8,000 rpm at 24°C for 10 minutes. The supernatant was recovered and 0.6 volume of cold isopropanol was added. The mixture was mixed by inverting the tuber for several times and centrifuged again as described above. Precipitated DNA was washed with ethanol (70%) and, after air drying, suspended in 500 *µ*l of TE buffer having the composition of 10 mM Tris (pH 8.0) and 1 mM EDTA. RNAse A was added and incubated at 37°C for overnight to remove any RNA contamination. The purity and integrity of DNA were evaluated by spectrophotometric method and agarose gel electrophoresis.

### 2.2. PCR, Cloning, and Sequence Analysis of Basrai TLP Gene

Primers for Basrai thaumatin-like antifungal gene were prepared on the basis of nucleotide sequence of TLP protein as described reported by Barre et al. [15]. The primer sequences were forward primer: 5′-GGTCCAGCATCCTCTCCCTC-3′; reverse primer: 5′-TCAGAAGAACCGGGAGTAGT-3′. TLP gene was amplified in a thermal cycler (Bio-Rad, Inc., USA) using the following parameters: initial denaturation at 94°C for 3 min, following 35 denaturation cycles at 94°C for 35 sec, annealing at 60.3°C for 35 sec, extension at 72°C for 35 sec, and then final extension at 72°C for 10 min. The PCR products were analyzed on 1.5% agarose gel and purified by GeneJET™ Gel Extraction kit K0691 (Fermentas). Ligation of purified PCR product was carried out into pTZ57R/T vector by using InsT/Aclon™ PCR cloning kit (K1214, Fermentas) and competent cells of* E. coli* DH5*α* were transferred with the ligation mixture. pTZ57R/Basrai TLPs containing recombinant plasmid positive clones were screened by blue-white screening method, colony PCR, and restriction analysis. The constructs containing plasmids were prepared according to Sambrook et al. [16]. Purified plasmid was digested with* Xba*I and* Bam*HI at 37°C for 16 hours to release the Basrai TLP gene fragment. The gene insert was subcloned into the pET22b(+) expression vector and competent cells of* E. coli* DH5*α* were transformed. The positive clones were established by colony PCR, restriction pattern, and sequence analysis of the cloned Basrai TLP gene. Sequence analysis and multiple sequence analysis were performed by using BLASTp at NCBI (https://www.ncbi.nlm.nih.gov/) and ClustalOmega (http://www.clustal.org/omega) programs.

#### 2.2.1. Expression and Optimization of Expression Conditions

The recombinant plasmid (pET22b-Basrai TLP) was transferred into* E. coli* BL21 (DE3) and consequently culture was spread on LB agar plates having composition of 1.0% tryptone, 0.5% yeast extract, 1.0% NaCl (pH 7.5), and ampicillin (100 *µ*g/ml). From LB agar plate, colony harboring recombinant plasmid was inoculated in 10 mL LB medium containing ampicillin at the concentration of 100 *µ*g/ml and incubated at 37°C for overnight. One ml seed culture was used further to inoculate expression culture containing selective antibiotic. To appraise the impact of IPTG on induction, the recombinant* Escherichia coli* BL21 (DE3) cells were grown at 37°C till the absorbance reached up to 0.5–0.8 before induction of expression. 0.5 mM IPTG was added and incubated for 4 hours further for protein expression. Cells, after centrifugation at 12,000 rpm at 4°C for 5 minutes, were resuspended in lysis buffer containing 50 mM Tris-Cl (pH 8.5), 100 mM NaCl, 1 mM PMSF, and 5 mM EDTA and sonicated at 1 minute interval at 25 pulses on ice with a power level fixed between 4 and 5. The extract was obtained by centrifuging the cells at 8,000 rpm for 15 minutes to isolate soluble and insoluble protein fractions. Both fractions were subjected to 15% SDS-PAGE according to the method of Laemmli [17].

Protein expression was optimized at different conditions such as effect of time, IPTG concentration, and autoinduction with lactose. To study the effect of time period on expression of Basrai TLP, transformed* E. coli* cells were induced by supplementing the medium with 0.5 mM IPTG. 1 ml culture samples were collected at 2-hour interval up to 12 hours. Collected samples were centrifuged at 10,000 rpm for 5 min and the pellets were resuspended and then analyzed by SDS gel electrophoresis. To optimize IPTG concentration, transformed cells were induced in the presence of different IPTG concentrations ranging from 0.1 to 0.9 mM. The cells were collected as described earlier, suspended, and examined on 15% SDS gel. Autoinduction studies were carried out with lactose as an inducer. Therefore, overnight cell culture was refreshed in 100 ml LB medium containing 100 *µ*g/ml ampicillin, 10 Mm lactose, and 20% glucose. Pre- and postinduction culture samples were collected as described above and ultimately examined by SDS gel electrophoresis.

#### 2.2.2. Western Blot Analysis and Antifungal Activity

For western blot analysis studies, antibodies were produced against purified Basrai TLP. Albino mice weighing 20–25 g were injected with the mixture of antigenic protein and Freund's complete adjuvant (FCA) in the ratio of 1 : 1. 100 *µ*l of total blend was injected and four times immunization was done at 10 days interval. After 40 days, antisera were drawn by cardiac rupture. Three booster doses were given and entire immunization procedure was performed in triplicate. Blood samples were drawn and antisera obtained were subjected to 35% ammonium sulphate precipitation at 4°C to remove nonspecific epitope impurities. Antisera were purified by Pearl, IgG purification kit (G-Biosciences, Cat# 786–798), and purified IgG was preserved at −80°C. Protein samples were fractionated by 15% SDS-PAGE and transferred on cellulose membrane by using Bio-Rad Trans-Blot. The membrane was blocked and then incubated at 4°C overnight with raised purified primary antibody prepared at 1 : 500 dilutions in TBS-Tween-20. Blot was incubated with AP-conjugated secondary antibody (1 : 5000 dilutions in TBS-Tween-20) for 2 hours at room temperature. Finally, the blot was developed in alkaline phosphatase (AP) substrate solution containing freshly prepared BCIP (5-bromo-4-chloro-3-indolyl phosphate, Sigma) and NBT (nitroblue tetrazolium, Sigma).

#### 2.2.3. Synergistic Assay

The term synergy describes the increase of antifungal activity of certain antibiotics by certain plant proteins termed as SAFPs which synergize antifungal antibiotics. In synergistic compositions, minimum inhibitory concentration (MIC) value of an antibiotic in the composition is smaller than the MIC value of the antibiotic in the absence of SAFP protein. For many human pathogens, such synergistic compositions of plant antifungal proteins and antibiotics are found to be very effective. Synergism is calculated by comparing MIC value of an antibiotic with that of MIC of antibiotic/protein composition. Antibiotic synergy plate method was used to determine the SAFP activity.

The fungal strains were grown on malt extract agar medium (MEA) for 7 days at 35°C. The spore suspension was prepared by washing slant surface with sterilized saline solution containing 0.2% Tween 20, counted by microscope, and then adjusted to 2 × 10^6^ conidial spores/ml. 100 *µ*l of cells suspension was added to the MEA at a temperature of 42°C to 45°C and 25 ml of this medium was added in each Petri plate.

Well diffusion method using the Kirby-Bauer technique [18] was used to perform antifungal susceptibility test of fungal strains. Combinations of antifungal TLP protein and antibiotics were employed under the guidelines of the National Committee for Clinical Laboratory Standards (NCCLS). Basrai antifungal protein at 2 and 4 mg/ml concentration and amphotericin B at 10 and 20 *µ*M concentrations was used against* A. fumigatus* alone and in blend. Amphotericin B alone and the mixture of Basrai TLP and antibiotic were poured into the wells made in MEA plates and assays were run in duplicate. Diameters of inhibition zones were measured to the nearest point where a prominent reduction in growth was obtained after 48 h.

#### 2.2.4. Structure Modeling

Modeling of thaumatin-like protein was carried out using the docking server, PatchDock, and further refined the docking results by FireDock.

#### 2.2.5. Antifreezing Activity Assay

Antifreeze proteins (AFPs) or ice structuring proteins (ISPs) are produced by different organisms. TLPs as well as other antifreeze proteins are found to adsorb on the surface of ice crystals therefore inhibiting the binding of additional water molecules creating an ice crystal surface having highly curved fronts and elevated surface free energy [19]. Subsequently, the growth of these fronts is ceased because the binding of water molecules to this surface is energetically less favorable. Further, the temperature needs to be lowered to reduce the free energy before crystal growth ensues and as a result, the freezing point of the solution drops. As an antifreeze agent, the protein helps to control the formation of ice crystals in various frozen foods like frozen yogurt, ice cream, different milk shakes, and so on. The presence of such antifreeze proteins in these frozen products may prevent growth of ice crystal and therefore preserve the smooth and creamy consistency of a high quality food product. To determine antifreeze activity of Basrai TLP, the frozen yogurt was prepared by using the following ingredients (weight in %): condensed skim milk 12.0; cream 7.0, ultrafiltered skim milk 33.0; corn syrup 11.0, sucrose 13.0; strawberry 1.0 and water 7.0.

### 2.3. Frozen Yogurt Preparation

Condensed skim milk, ultrafiltered skim milk, and cream were mixed with each other to obtain fat content up to the desired level. After homogenizing the milk, other ingredients were added and heated before culture inoculation. The commonly used temperature and time duration for yogurt preparation is 85°C for 30 minutes or 90–95°C for 5 minutes. The temperature treatment is required to destroy unwanted microorganisms from the preparation. Later, the milk was cooled to a temperature prerequisite to incubate the culture. An optimum temperature of 40–45°C is required for the fermentation of* Lactobacillus delbrueckii*. During fermentation process, lactose is converted into lactic acid resulting in the lowering of pH (4.6) where gelation takes place. When the desired pH of the yogurt obtained and yogurt partly cooled to 20°C, the strawberry was added. Now the yogurt was separated into two parts; in one portion Basrai TLP protein was added at the concentration of 10 ppm and other portion was used as a control. Finally, the yogurt products were blast frozen and then stressed for four days before examination [20].

#### 2.3.1. Microscopic Analysis

To analyze ice crystals, a small amount of freezing sample was placed on the cold microscope slide with cold spatula. Zones where ice crystals uniformly distributed and separated were selected to examine ice crystals by image analysis.

## 3. Results and Discussion

Pathogenesis-related PR5 proteins are known as thaumatin-like proteins (TLP) due to their amino acid sequence homology with thaumatin and have been isolated from a number of crop plants such as wheat, corn, rice, and soybeans [6, 7]. Studies have shown that these pathogenic proteins are one of the important members of PR families which play vital role in plant defense system against various pathogens.

Thaumatin-like proteins are present in not only vegetative tissues but fruits of different dicots as well. Large amounts of PR-5 proteins accumulate in ripening process and fruit specific PR-5 proteins with antifungal activity have a significant role in fruit maturation [13].

### 3.1. Molecular Cloning

**Figure 1:**
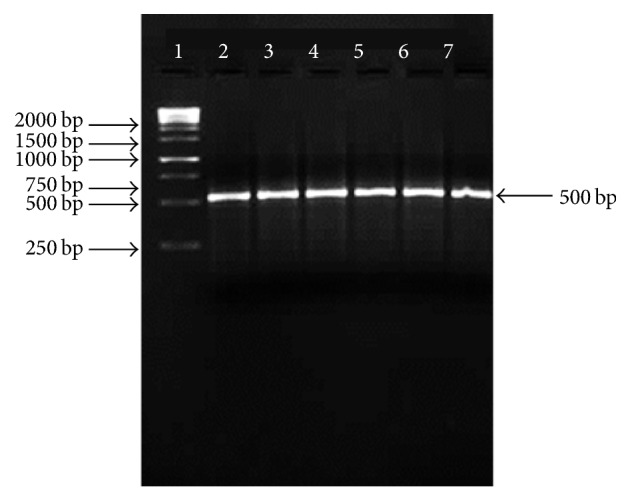
PCR amplified product of Basrai TLP observed on 1.5% agarose gel. Lane 1: 1 kb DNA ladder (Fermentas # SM1163); Lane 2–7: basrai TLP amplified products.

**Figure 2:**
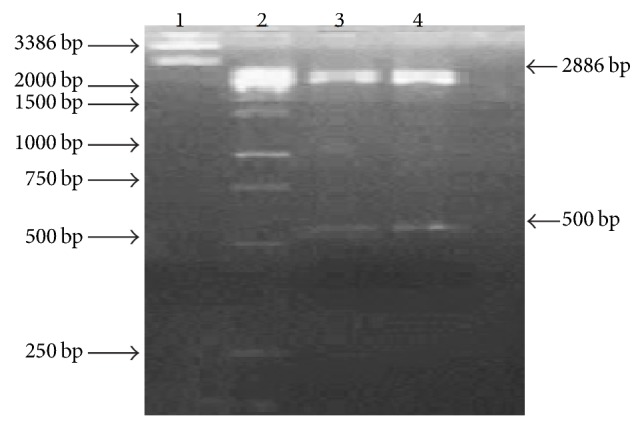
Restriction analysis of pTZ57R/Basrai TLP plasmid. Lane 1: pTZ57R/Basrai TLP plasmid; Lane 2: 1 kb molecular weight DNA ladder (Fermentas #SM1163); Lane 3-4: pTZ57R/Basrai TLP plasmid restriction with* Bam*HI and* Xba*I.

**Figure 3:**
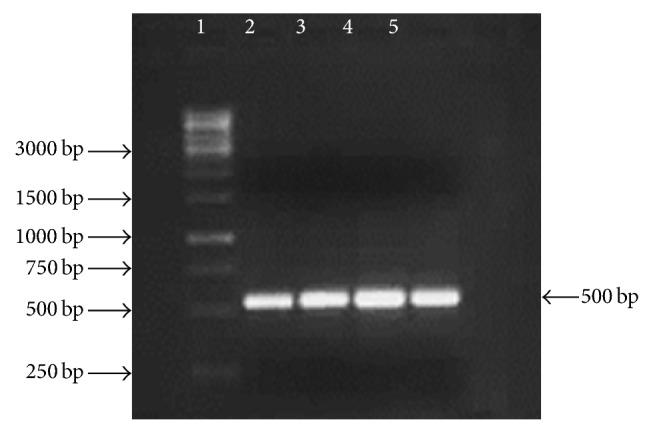
Colony PCR of* E. coli* DH5*α* colonies transformed with pET22b-Basrai TLP. Lane 1: 1 kb molecular weight DNA ladder (Fermentas #SM1163); Lane 2–5: colony PCR of Basrai TLP fragment.

Putative TLP gene encoding an antifungal protein from Basrai banana was amplified from genomic DNA with PCR by using specific primers. About 500 bp product was amplified (Figure 1) and the molecular size of Basrai TLP gene was in accordance with the anticipated molecular weight of other homologue TLPs [15]. The purified PCR product was cloned into pTZ57R/T vector and subsequent pTZ57R/Basrai TLP construct was used to transform the competent cells of* E. coli* DH5*α*. Positive transformants were selected by blue-white colony selection, colony PCR, and restriction analysis with* Bam*H1 and* Xba*1 restriction enzymes (Figure 2). The Basrai TLP gene fragment harbored in pET22b(+) was expressed in* E. coli* DH5*α* cells and resulting positive clones were selected by colony PCR (Figure 3).

**Figure 4:**
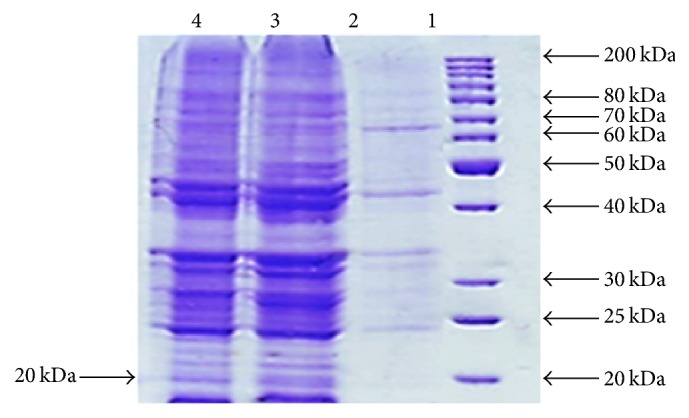
15% SDS-PAGE of total protein of* E. coli* BL21 codon Plus transformed with pET22/Basrai TLP.

For expression of basari TLP,* E. coli* BL21 (DE3) cells were transformed with recombinant pET22b-Basrai TLP and resulting expression cells were grown in LB medium and further induced in the presence of IPTG inducer. The optimized conditions, induction with 0.5 mM IPTG and 6 hours incubation at 37°C, were used to express protein in* E. coli* BL21 (Figure 4). Large amount of protein inclusion bodies were found in insoluble protein fraction of transformed* E. coli* BL21 cells. The inclusion bodies are often observed when proteins are overexpressed. Faus et al. [21] have reported similar results when thaumatin protein from* T. daniellii* was expressed in* P. roqueforti* and recombinant protein with molecular weight of 25 kDa was found as inclusion bodies. Hu and Reddy [22] also observed inclusion bodies of thaumatin-like protein (ATLP3) from* Arabidopsis* when expressed in* E. coli *and also found that refolded protein displayed antifungal activity against certain fungal pathogenic strains. Newton and Duman [23] expressed osmotin-like cryoprotective protein from* S. dulcamara* in* E. coli* and soluble protein fraction was found in abundance in periplasmic compartment. On the other hand, when this protein was expressed in cytoplasmic space, aggregated and insoluble protein was released in great quantity. It is reported that protein inclusion bodies, protein, and amyloid aggregates are formed as a result of protein accumulation which may consist of misfolded proteins as well [24].

**Figure 5:**
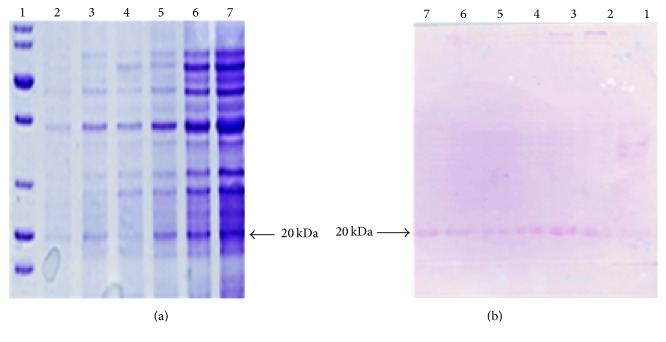
SDS-PAGE and Western blot analysis of Basrai TLP protein. (a) SDS-PAGE, Lane 1: protein ladder (Fermentas #SM0661); Lane 2–7: cells were induced with 0.5 mM IPTG and collected after 2, 4, 6, 8, 10, and 12 hours of induction, respectively. (b) Western blot analysis, proteins from gel were transferred onto nitrocellulose membrane and immunostained with mouse anti-plant Basrai TLP antibody.

In our study, maximum recombinant protein expression was obtained at 0.7 mM concentration of IPTG at 37°C after 6-hour incubation. Donovan et al. [25] have reported a range of IPTG concentration (0.005–5 mM) which can be used for inducing gene expression and it is also noted by Glick [26] that generally high concentrations of IPTG may not necessarily effect the maximal recombinant protein expression. Maximum protein was expressed after 6-hour incubation in the presence of lactose as an inducer and time course studies. About 20 kDa protein was observed after western blot analysis of recombinant proteins expressed in the induced cells of* E. coli* BL21 (DE3) as shown in Figure 5.

### 3.2. Synergistic Assay

**Figure 6:**
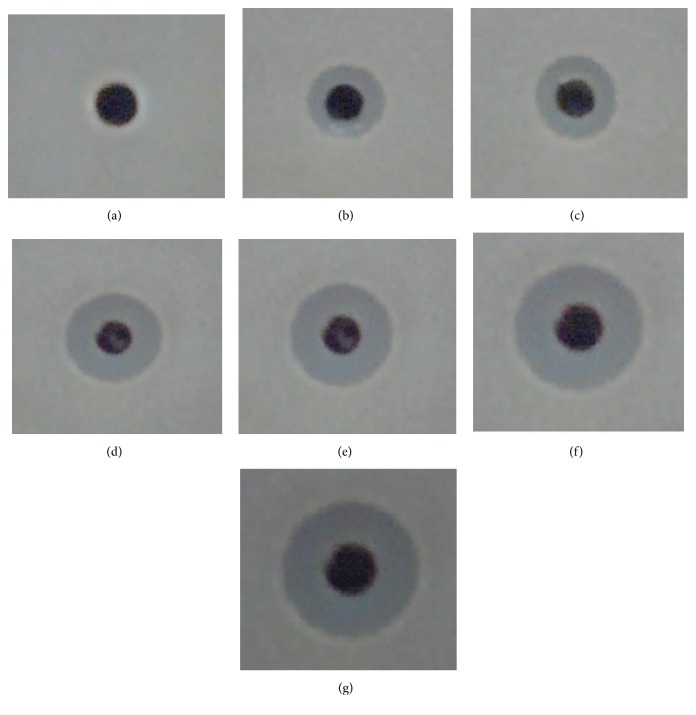
Synergistic assay of purified Basrai antifungal protein with amphotericin B against* A. fumigatus*. (a) Control; (b) 2 mg/ml Basrai antifungal protein; (c) 4 mg/ml Basrai antifungal protein; (d & e) amphotericin B with concentrations 10 and 20 *µ*M; (f & g) combination of Basrai antifungal protein and amphotericin B.

The potential of antifungal TLP as pharmaceutical agent for the management of human and animal fungal diseases was assessed by measuring the inhibition zones in agar well diffusion method against* A. fumigatus*. For this purpose, two doses of 2 and 4 mg/ml of Basrai antifungal protein and 10 and 20 *µ*M of amphotericin B were tried against* A. fumigatus* individually and in blend as well. Antifungal TLP exhibited inhibition zones of 4.0 and 5.0 mm diameter against* A. fumigatus* when used at the concentration of 2 and 4 mg/ml while 6.0 and 7.0 mm inhibition zones were found with amphotericin B at 10.0 and 20.0 *µ*M concentrations against tested strain. Though basrai TLP and amphotericin B independently exhibited their antifungal potential against* A. fumigatus,* however, their combined effect enhanced their activity as shown by increase in diameter of inhibition zones (Figure 6). Stevens et al. [27] have already established a synergistic relation between zeamatin and nikkomycin Z antibiotic, an inhibitor of chitin synthesis in fungal cell wall, and with clotrimazole antibiotic, which prevent membrane sterol synthesis [28].

### 3.3. Structure Modeling

Odds et al. [29] have stated different target areas of fungal strains for the action of various antifungal agents. Major focused targets of these antifungal targets are their cell wall and plasma membrane components, mainly membrane sterol, ergosterol, and their synthesis. Mesa-Arango et al. [30] have described a mechanism by which amphotericin B produces toxicity against fungal infections. The drug binds with ergosterol component of fungal cell wall and results in the formation of pores through which different essential anions and cations leak out and ultimately cause fungal cell death. After apprehending the inhibitory effect of antifungal agents, docking studies were carried out to evaluate the antimicrobial potential of TLP against* A. fumigatus* by probing the binding pattern of antifungal protein with membrane ergosterol of targeted microbial strain and simultaneously docking of amphotericin B with* A. fumigatus* membrane ergosterol was also investigated.

Basrai TLP, ergosterol, and amphotericin B were imported to PatchDock server and docked together. Clustring RMSD was given as 4.0A and docking job was submitted to PatchDock server. Followed by this the protein to ligand docking solutions obtained from PATCH dock server were further refined by FireDock which rendered additional refinements to docking complexes. FireDock server analysis sorts out the refined complex structures based on energy functions like global energy, atomic contact energy, contribution of the hydrogen bond, Van der wall interactions, and so on.

**Figure 7:**
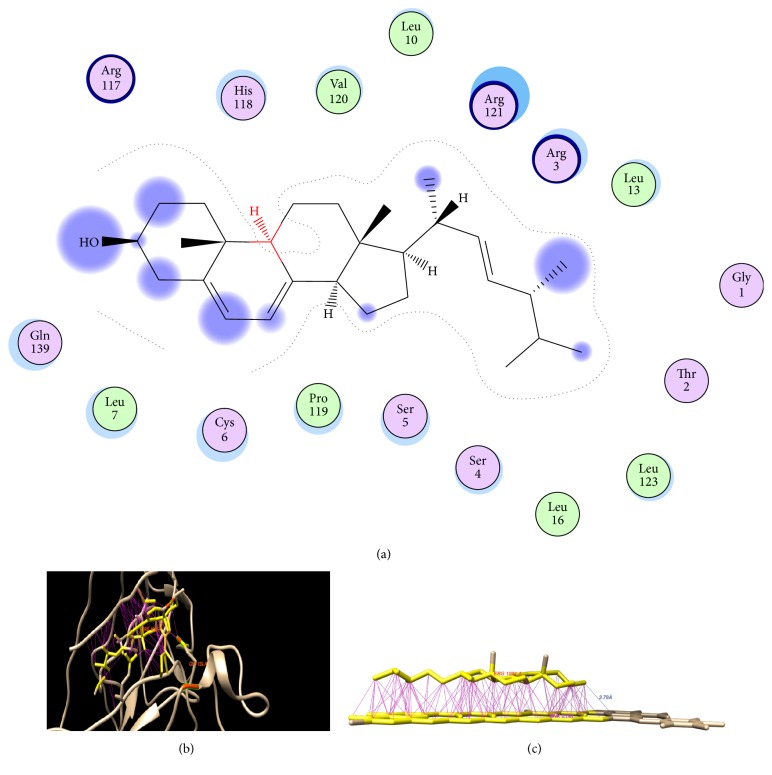
Molecular docking interaction. 2D illustration of ergosterol with the binding sites of TLP (a & b); 2D illustration of amphotericin B with ergosterol (c).

Amino acid residues, that is, Arg117, His118, Val120, Arg121 and Arg3, Leu13, Gly1, Thr2, Leu123 and Leu16, Ser4 and Ser5, Pro119, Cys6, and Leu7 of TLP were found to involve in binding with membrane ergosterol through* van der Waals* interactions; however, Gln 139 amino residue interacts with the ergosterol through hydrogen bonding pattern as shown in Figure 7. The study proposes that basari TLP has high binding affinity towards plasma membrane ergosterol of* A. fumigatus* and potentially induces damage in the cell membrane of targeted pathogenic fungal strain.

Docking studies of amphotericin B with ergosterol also demonstrated their hydrogen bonding and van der Waals interactions as shown in Figure 7. The results may indicate that both basari TLP and amphotericin B interact with membrane ergosterol hence causing fungal cell lysis therefore supporting our experimental study that blend of amphotericin B and basari TLP greatly amplified the antifungal activity against* A. fumigatus* pathogen.

The bananas are available throughout the year and locally grown in Pakistan and according to Marr et al. [31]* A*.* fumigatus* is responsible for most of clinical manifestations related to fungal infections therefore the production of Basrai antifungal protein may exploit its fungicidal or fungistatic aspect and produce cost effective drugs at large scale. Therefore, this finding opens up new avenues of research and indigenous substitutes for imported and expensive drugs in the field of medicine.

### 3.4. Basrai Antifungal TLP as Antifreezing Agent

Besides evident role of TLPs against pathogenic microbes, some TLPs in plants are found to be associated with their adaptation to winter environments. Kuwabara et al. [32] have reported a related TLP from wheat source showing its low temperature activity in addition to antifungal potential. According to Yu and Griffith [19], antifreeze activity of TLPs plays its role in inhibiting intercellular ice formation and recrystallization in cold tolerance twigs. The quality of freezing foods declines due to the formation of large ice crystals when exposed to low temperature for their long time storage and according to literature antifungal proteins are involved in preventing ice crystallization; therefore these proteins may find their interesting role in frozen food industry [33].

**Figure 8:**
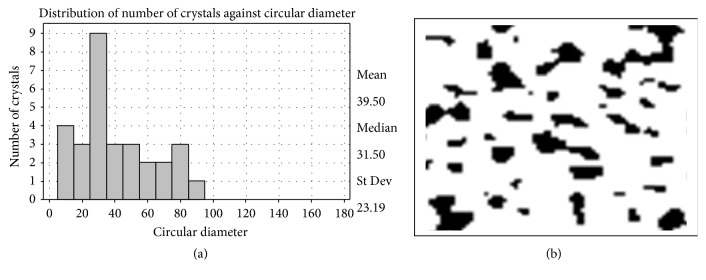
Distribution of ice crystals in frozen yogurt without Basrai TL hardened at −40°C and stressed at −29°C for 4 days and (b) micrograph of ice crystals in frozen yogurt without Basrai TL hardened at −40°C and stressed at −29°C for 4 days.

**Figure 9:**
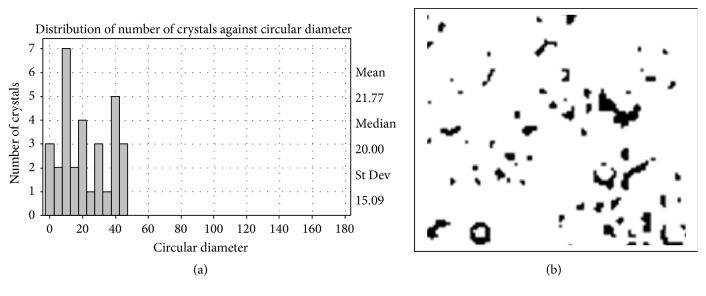
(a) Distribution of ice crystals in frozen yogurt with Basrai TL slowly frozen at −25°C and stressed at −29°C for 4 days and (b) micrograph of ice crystals in frozen yogurt with Basrai TL slowly frozen at −40°C and stressed at −29°C for 4 days.

In the present study, the potential of Basrai antifungal TLP as antifreezing agent in frozen yogurt was explored because so far bananas have not been considered for such perspective application. The antifreeze activity was analyzed at different freezing conditions and size and number of crystals were measured. Large size crystals were observed in frozen yogurt without Basrai antifungal protein which was hardened at −40°C and stressed at −29°C for 4 days (Figures 8(a) and 8(b)). The small size ice crystals were, however, observed in frozen yogurt with Basrai antifungal protein, frozen very slowly at −40°C for 16 hours, and stressed at −29°C for 4 days (Figures 9(a) and 9(b)). As an antifreezing agent the protein assists in controlling the ice crystal formation in frozen yogurt. Basrai antifungal protein was found to reduce the size of ice crystals and making it flatter and softer in taste even after being refrigerated. Its presence in frozen yogurt inhibited ice crystal growth and preserved the smooth and creamy texture of yogurt. Basari TLP antifreeze protein is a good contribution to the list of such agents already being used for this purpose. Some of these antifreeze agents used in various foodstuffs are obtained from fish source which eventually affects the cost of such agents. Although basari TLP as antifreezing agent is just an addition, its cost effective aspect is undeniable as mentioned above that bananas are one of such food items which are readily and continuously available without time or seasonal constraint. So if they are considered in this context, bananas might be exploited in iced food items.
